# Effects of a Dialogic Book-sharing Intervention for Female Caregivers in Rural Tanzania (EDBiT): Protocol for a Randomized Controlled Trial

**DOI:** 10.2196/68758

**Published:** 2025-09-08

**Authors:** Martin Schlossarek, Eva Šerá Komlossyová, Lynne Murray, Peter J Cooper, Ondřej Vencálek, Lenka Dušková, Maregesi Machumu, Simona Šafaříková, Jaromír Harmáček, Miroslav Syrovátka

**Affiliations:** 1 Department of Development & Environmental Studies Palacký University Olomouc Olomouc Czech Republic; 2 School of Psychology and Clinical Language Sciences University of Reading Reading United Kingdom; 3 Department of Mathematical Analysis and Applications of Mathematics Palacký University Olomouc Olomouc Czech Republic; 4 Department of Educational Psychology and Curriculum Studies Dar es Salaam University College of Education Dar es Salaam United Republic of Tanzania

**Keywords:** dialogic book-sharing, cognitive development, child language, parenting, Tanzania, study protocol

## Abstract

**Background:**

Children in low- and middle-income countries face obstacles to optimal language and cognitive development due to a variety of factors related to adverse socioeconomic conditions. One of these factors is compromised caregiver-child interactions and associated pressures on parenting. Early development interventions, such as dialogic book-sharing (DBS), address this variable, with evidence from both high-income countries and urban areas of low- and middle-income countries showing that such interventions enhance caregiver-child interaction and the associated benefits for child cognitive and socioemotional development. Yet, evidence for DBS effects is lacking from poor rural communities where the need for such early development intervention may be greatest.

**Objective:**

The objective of this study is to assess the effects of a DBS intervention, a parenting program for female caregivers of children aged between 15 and 45 months, implemented in rural Tanzania. We aim to assess the impact of the intervention on the following domains: child cognitive and socioemotional skills, parenting and parental stress, and child health.

**Methods:**

The study is a 3-arm cluster randomized controlled trial. In total, 443 female caregivers participated in the study. Clusters of caregivers were randomized to either (1) an index DBS intervention group, (2) a playful activity active control group, or (3) a waitlist control group. The active control group was designed to control for any attention effects, ensuring that observed improvement in the index group can be attributed to the DBS intervention’s content. The primary outcomes were child language, parental sensitivity, and parent-child interaction. The secondary outcomes concerned child attention and behavior, parenting practices, and parental stress. A combination of questionnaires and direct observations was applied. Qualitative methods were also used, primarily to capture caregivers’ experiences and subjective perspectives on intervention-induced changes.

**Results:**

Data collection for the study was completed in September 2024. The study results are expected to be published by late 2025.

**Conclusions:**

This randomized controlled trial of a DBS intervention implemented in rural Tanzania adds to a growing body of international literature exploring the impact and limitations of a simple and scalable early development intervention to enhance child outcomes.

**Trial Registration:**

International Standard Registered Clinical/Social Study Number (ISRCTN) ISRCTN12613329; https://www.isrctn.com/ISRCTN12613329

**International Registered Report Identifier (IRRID):**

DERR1-10.2196/68758

## Introduction

### Background

Children from low- and middle-income countries (LMICs) face multiple challenges that significantly impede their cognitive and socioemotional development [1]. These challenges pose a substantial threat to their well-being in adulthood. Children’s early language and cognitive development, at risk in such contexts, are important determinants of subsequent literacy skills and later school progress [2,3].

Several socioeconomic conditions and poverty-related factors contribute to compromised child developmental progress [4-6]. These include nutritional deficits, which directly impact both physical and cognitive growth; limited access to educational resources, such as learning toys; elevated risk of chronic stress exposure; and the challenges posed by overcrowded and inadequate housing conditions. All of these disrupt the educational process [1,7] and children’s health status [8,9].

Compromised caregiver-child interaction and limited caregiver activities that support early learning are other key factors prevalent in deprived contexts that hinder children’s cognitive and socioemotional development [6,10,11]. Early difficulties in the caregiver-child relationship raise the risk of insecure infant attachment [12], itself predictive of a range of adverse child outcomes [13]. In addition, promoting warm and responsive interactions among caregivers, including vocalizations and smiles, has been shown to positively influence child health outcomes [14].

Providing education to parents in dialogic book-sharing (DBS) techniques has been proposed as a simple and deliverable means of obviating adversity risk to the caregiver-child relationship and child development [15,16]. There is considerable evidence for the benefits of book-sharing interventions provided to families living in high-income countries [16-19]. While a similarly beneficial impact has been reported from urban and periurban areas of LMICs [15,20-22], evidence is limited from poor rural communities, where the need for such early child development intervention may, arguably, be the greatest [10,23,24].

This paper presents the protocol for the Effects of a DBS Intervention for Female Caregivers in Rural Tanzania (EDBiT) study. EDBiT is a randomized controlled trial (RCT) designed to determine the effects of the DBS intervention delivered in rural Tanzania on cognitive development (specifically language skills and attention) as well as other parental and child outcomes.

During the preparation phase of the trial, we paid particular attention to the five recommendations proposed by Noble et al [18] to increase the probability that education in DBS will benefit the targeted child outcomes: (1) The DBS intervention delivered in this study involved a higher dosage compared to most previous studies [10,16,18,20-22,25], as the core 8-week educational program (intensive intervention) was followed by regular meetings of female caregivers swapping books and exchanging experiences with book-sharing and the upbringing of children (extensive intervention). (2) In addition to the waitlist control group, we included an active control group to test specific effects of book-sharing over the nonspecific effects of being subjected to some educational program focused on some parenting skills. (3) The design of this trial allows a potential follow-up that will assess the long-term effects of the index intervention. We expect the data collection for this follow-up study to be conducted in 3- to 5-year intervals, with the theory of change (ToC), presented in the Overview of the Study Design section, determining variables that will be the focus of our interest. (4) We collected data on a broad range of outcome variables to provide a rich assessment of the effects of book sharing on a variety of outcomes. (5) Children from diverse socioeconomic backgrounds living in the target region were included, allowing for the investigation of moderating social factors.

### Aims

The aim of the EDBiT study is to assess the effects of DBS, a parenting program for female caregivers of children aged between 15 and 45 months, implemented in rural Tanzania. We aim to assess the impact of the intervention on the following domains: child cognitive and socioemotional skills, parenting and parental stress, and child health. We will evaluate the moderating influence of certain variables, including caregivers’ cognitive abilities.

## Methods

### Overview of the Study Design

This study is a 3-arm cluster RCT. Registered female caregivers were randomized at the individual level to an index intervention group attending DBS educational sessions, an active control group attending educational sessions focused on playful activities (PAs) designed to promote the development of a child’s motor skills, or a waitlist (passive) control (WLC) group. We collected data on all 3 groups at baseline, after intensive intervention, and after extensive intervention, approximately 1 year after the start of the intervention (refer to the Intervention section for the description of the intensive and extensive phases of the intervention).

On the basis of previous research on the efficacy of the version of DBS implemented in this study [15,20-22], significant differences between the index group and the WLC group are to be expected. The comparison between DBS and PAs is to control for the fact that any parenting interventions may yield positive results for children because of the caregivers’ increased attentiveness to their children rather than because of the content of the index intervention itself. While DBS primarily aims to develop child language, PAs were strategically designed to cultivate other skills that are not the primary focus of DBS (refer to the subsequent sections).

Several authors argue for adding qualitative methods to quantitative ones in RCTs for studies to paint a richer picture of the evaluated interventions and increase the validity of findings [26-28]. However, most existing RCTs of DBS programs apply solely quantitative methods [17,18,20-22]. In EDBiT, we adopted a mixed methods approach, with qualitative research capturing the subjective perspectives of caregivers in both DBS and PAs on the intervention’s effects. Qualitative data will provide an additional layer of information with the potential to further explain the results of the quantitative analysis, possibly shedding light on the factors that facilitated or hindered the realization of intervention outcomes. They will also examine how the intervention influenced caregivers’ parenting practices and attitudes toward their role in a child’s development. Caregivers’ book-sharing patterns were monitored through self-completed diaries and interviews. The level of parental engagement could moderate the index intervention’s impact on child outcomes [18].

**Figure 1:**
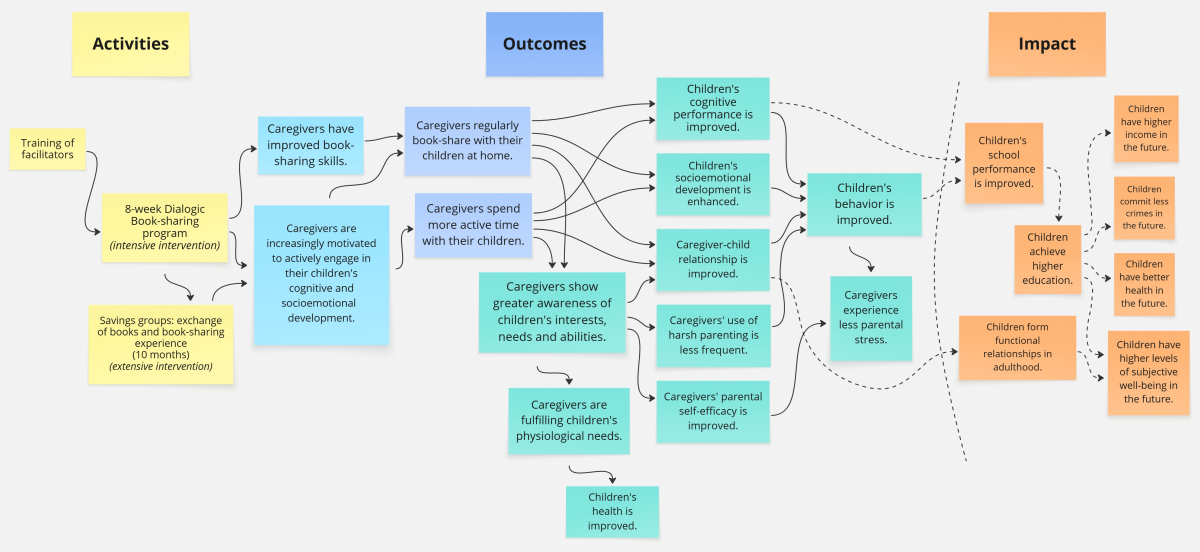
Theory of change of the dialogic book-sharing intervention.

Considering the importance of theories of change for impact evaluations [29], we constructed the causal chain (Figure 1). The model includes the main activities of the evaluated intervention, the intended immediate outcomes, and an indicative list of expected long-term impacts (beyond the scope of this study).

### Study Setting

This study has been conducted in the rural areas of the Usangu Plain, South West Tanzania. Specifically, it is located along the southern and eastern edges of the Usangu area, historically labeled as the “cultivation zone” [30]. This populous area relies on small-scale farming, mainly on the production of rice and maize. Implementing the EDBiT study in Usangu provides an opportunity to test the feasibility and effectiveness of a DBS intervention in a context that is close to the socioeconomic median for Africa rather than at either extreme. The Multidimensional Poverty Index places both the South West Highlands region, where Usangu is situated, and Tanzania as a whole near the median of their respective national and continental distributions [31]. Findings from such a setting may offer valuable insights into the potential scalability of the DBS program elsewhere across the African continent.

**Figure 2:**
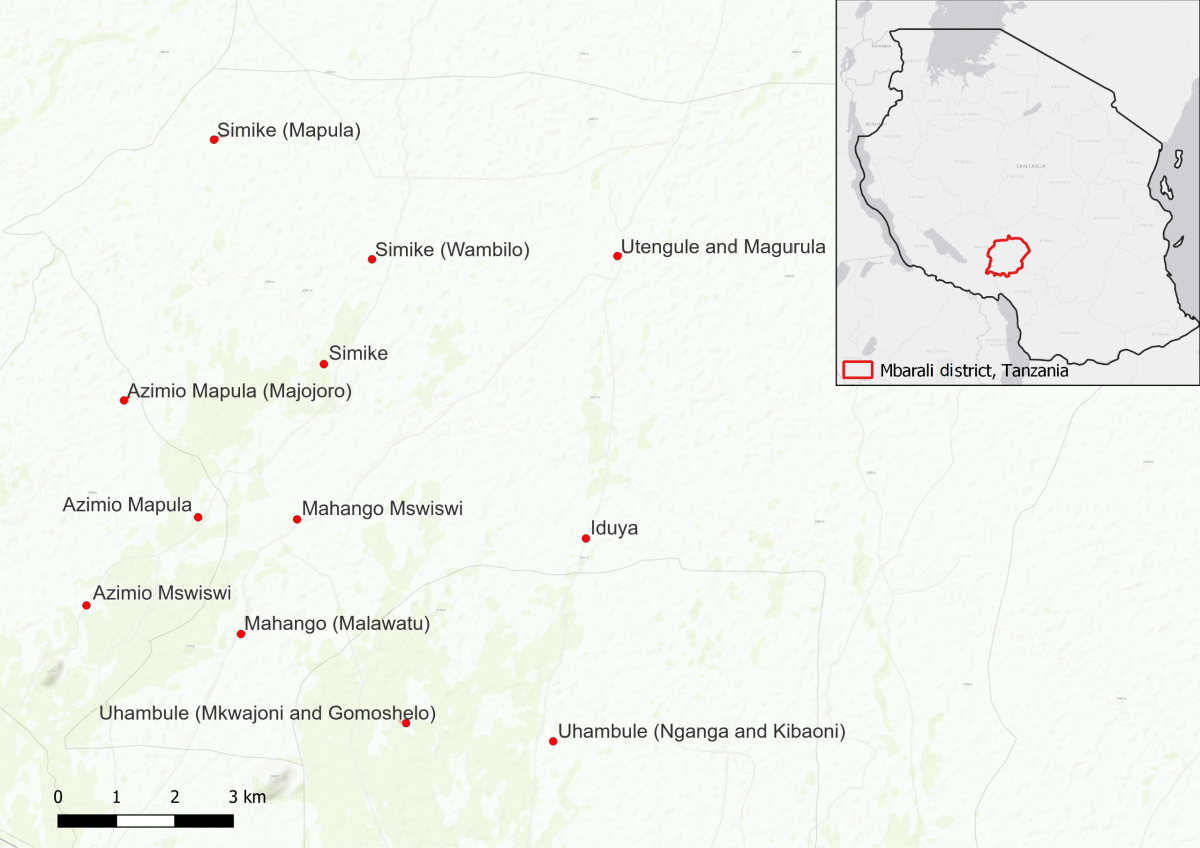
Location of the Effects of a Dialogic Booksharing Intervention for Female Caregivers in Rural Tanzania study villages. Produced by the authors in QGIS (Version 3.40) using World Topo Map (Sources: Esri, DeLorme, HERE, TomTom, Intermap, increment P Corp., GEBCO, USGS, FAO, NPS, NRCAN, GeoBase, IGN, Kadaster NL, Ordnance Survey, Esri Japan, METI, Esri China (Hong Kong), swisstopo, MapmyIndia, and the GIS User Community) [32] and Light Gray Canvas Map (Sources: Esri, DeLorme, HERE, MapmyIndia) [33]. Individual village data sourced and included by the authors.

This study covers 12 towns, villages, and geographically isolated neighborhoods (referred to as “villages” in Figure 2) from Utengule Usangu, Kongolo, and Igurusi wards (Figure 2) [32,33]. Assessment and education sessions were held in each of the 12 villages. Village executive officers were informed about the project, and leaders in all villages approved both the intervention and the study.

### Ethical Considerations

This study received ethical approval from the Faculty of Science Ethics Committee at Palacký University Olomouc (reference 23-03) and from the University of Dar es Salaam (reference AB3/12B). Caregivers provided informed consent for their own and their children's participation after the full consent form was read aloud to them during registration, and again verbally prior to each instance of data collection. All consent was audio-recorded. Participation was voluntary, and caregivers were informed that they could withdraw at any time without consequences. No personally identifying information was collected, and all data were anonymized prior to analysis. Participants did not receive financial compensation, but small refreshments and gifts were provided as a token of appreciation. All the data are securely stored on a cloud server and made available only to the core research team members to maintain confidentiality. The study has been registered with the International Standard Registered Clinical/Social Study Number (ISRCTN12613329).

### Eligibility, Recruitment, and Randomization

Female caregivers caring for a child born between December 1, 2019, and June 1, 2022 (aged between 15 and 45 months at the time of baseline assessment), were eligible for participation in this study. Eligible women were the primary persons responsible for childcare and not necessarily biological mothers.

Recruitment of caregivers was conducted between July 29, 2023, and September 3, 2023. Members of the project team visited prospective households, accompanied by respected women from the local communities. Eligible caregivers were enrolled in the study if they (1) provided consent to participate in the study; (2) declared their availability to attend the 3 rounds of assessments; and (3) agreed that we could, if necessary, delay their participation in the project by 1 year.

In total, 542 eligible caregivers enrolled in this study. For each village, the location of each household was determined by GPS, and mutual distances between households were computed. To prevent cross contamination, we divided groups of households into clusters. To avoid large clusters of households, we selected a subset of households that could be split into smaller clusters of no more than 5 households. The distance between households from distinct clusters was set as >100 m. Some eligible households were excluded from the study to fulfill this condition. These caregivers were invited to join savings groups a year later (refer to the Intervention section). The number of excluded households (n=99) was kept to a minimum by using advanced graph theory methods. Therefore, 443 (81.7%) female caregivers were included in this study.

Clusters, each including 1 to 5 households, were randomly assigned to the DBS, PA, or WLC group. The randomization process mimicked a block design; it considered dwelling place (village) and child age (children aged between 15 and 30 months vs children aged >30 to 45 months) and ensured a balanced distribution of these characteristics across the study groups.

The study participants were not informed which program (DBS or PA) constituted the studied intervention and which served as a control condition.

### Intervention

#### The Index Intervention Group (DBS)

The index intervention was a DBS program consisting of 8 weekly educational sessions for groups of 3 to 8 female caregivers, each lasting 90 minutes [15,20,22]. This stage of the intervention is called the intensive intervention phase. The content of weekly sessions was based on the Mikhulu Trust book-sharing program for children aged 15 to 30 months and the Mikhulu Trust book-sharing program for children aged 30 to 45 months, developed by LM and PJC. These programs have been shown to benefit parenting and child development [20-22]. The program for caregivers of children aged between 30 and 45 months is described in Table 1.

**Table 1:** Summary of index intervention group sessions for caregivers of children aged between 30 and 45 months^a^.

Session number	Name of the session	Description of the session
1	Introduction to book sharing	Introducing basic dialogic book-sharing skills—following the child’s lead, using a lively voice, and setting up book-sharing routines
2	Pointing and naming	Basic dialogic book-sharing skills—pointing and naming, repeating and elaborating on things that interest the child, and finding opportunities for praise
3	Naming and linking	Asking “where, who, and what” questions, linking book content to the child’s own experience, and finding opportunities to use actions (eg, hugging and eating)
4	Talking about feelings	Helping the child understand the meaning of basic emotion terms—happy, sad, angry, and scared—and discussing why book characters feel the way they do, using facial expression and tone of voice to convey a character’s feelings, and linking feelings in the book to the child’s experience
5	Talking about intentions	Discussing why characters feel the way they do, asking what characters think and intend, and encouraging the child to be curious about what will come next in the story
6	Talking about perspectives	Helping the child understand that different people can see things differently, know different things, and feel differently about things
7	Summary	Reviews of key principles from sessions 1 to 6.
8	Graduation event	Certificates of completion presented to participants along with summary take-home cards and selected books from sessions 1 to 6; a group discussion about how to remain motivated about book sharing and how to access children’s books

^a^The program for caregivers of children aged between 15 and 30 months was broadly similar in content, with less emphasis on the components beyond these children’s developmental competence, such as perspective taking.

The sessions were delivered by women from the local community selected during the recruitment phase based on their previous experience working with mothers and small children. A 7-day DBS training with a certified Mikhulu Trust trainer was organized for 20 potential session facilitators. After the first 2 days of the training, the trainer selected the 10 best-performing women based on their book-sharing skills and presentation abilities demonstrated in class. These women completed the full facilitator training.

Supervision and experience sharing of intervention facilitators occurred during weekly meetings with the project team. In addition, the project team conducted ad hoc visits to sessions, observing the facilitators’ performance, ensuring session fidelity, and offering feedback. Each facilitator was visited twice throughout the intensive intervention phase.

Sessions followed a standard format. They began by reviewing participants’ experience of book sharing over the previous week. A presentation with Microsoft PowerPoint slides and instructional videos followed, introducing new book-sharing techniques. The session concluded with a discussion of “the book of the week,” that is, the book lent to parents for use over the following week. Children were looked after separately by a woman from the local community and given fortified porridge at every session to increase caregivers’ motivation to attend sessions. Caregivers were encouraged to participate in sessions via phone on the day before each session. At the end of the DBS program, all caregivers received a book to take home and keep.

A 10-month extensive intervention phase followed after graduation when caregivers were encouraged to meet biweekly to exchange books and discuss book-sharing and child-rearing experiences. Parents received an additional book 1 and 4 months after the end of the intensive intervention. To help sustain the motivation of caregivers to continue these meetings, we supported caregivers in forming charitable savings groups, locally named *simwabas*, with a 10-month saving cycle. During this phase, we sent short motivational messages encouraging participation in the meetings, practicing book sharing at home, and sharing feedback on book sharing and childcare with fellow caregivers. This aimed to further bolster parental motivation to sustain engagement with DBS.

#### The Active Control Group (PAs)

In the PA condition, female caregivers attended 8 weekly group-based educational workshops focused on PAs for children (intensive intervention phase). During the workshops, female caregivers in groups of 3 to 8 people were shown simple children’s games and how to produce low-cost toys using available materials to enhance children’s motor development. The program was appropriate for children aged between 15 and 45 months. The content of individual sessions developed by the research team is described in Table 2.

**Table 2:** Summary of active control group sessions (playful activities).

Session number	Content of the session	Description of the session
1	Introduction, puzzles, and gross motor skills exercise	This session includes introducing the program and explaining the relevance and benefits of the program for children’s development. Caregivers develop a set of puzzles and learn a game, enhancing gross motor skills.
2	Ring toss game and fine motor skills exercise	Caregivers produce rings for the ring toss game and learn a game, enhancing soft motor skills.
3	Coloring books and gross motor skills exercise	Caregivers create coloring books and learn a game, enhancing gross motor skills and nonverbal communication.
4	Dancing with children	Caregivers dance with their children while singing local music. Caregivers learn a game that helps children explore their senses and improve their attention.
5	Memory game and gross motor skills exercise	Caregivers produce a memory game and learn a game, enhancing gross motor skills.
6	Playing cards	Caregivers produce playing cards and learn a simple card game.
7	Dancing with children and playing cards	Caregivers dance with their children while singing local music. Then, they learn a simple card game.
8	Graduation event	Certificates of completion are presented to the participants. Groups discuss how to remain motivated to raise healthy children.

Local women, distinct from book-sharing facilitators, were trained in conducting PA sessions and supervised by the project team. This training was carried out on an ongoing basis, with weekly sessions dedicated to the upcoming lessons. Supervision and experience-sharing mechanisms mirrored those used in DBS.

PA sessions followed a standard format, starting with a review of the previous week’s activities. Caregivers then engaged in the main weekly activity, often involving the creation of simple toys. When possible, caregivers performed the activity with their children in the session. In addition, the facilitator suggested 1 more activity for caregivers to do with their children at home. Other session components mirrored those of the index intervention group.

After graduation, groups were encouraged to meet every 2 weeks for 10 months. This phase is analogous to the extensive intervention of the DBS condition. Instead of receiving picture books, caregivers were given written instructions twice during this period on creating other simple educational toys. We also supported them in establishing savings groups with a saving cycle of 10 months.

#### WLC Group

The passive control group was put on the waiting list to receive an intervention after completing the extensive intervention phase and the post–extensive intervention data collection. Caregivers in this group receive the service provided to the DBS condition 1 year after the baseline assessment.

### Study Assessments

#### Overview

Caregiver-child dyads from intervention and active and passive control groups underwent quantitative assessments at baseline, after intensive intervention, and after extensive intervention. The data collection team, comprising 10 local women and 4 Tanzanian team members, was trained and supervised by senior team members, including the study’s principal investigator (M Schlossarek), who ensured adherence to the protocol. We conducted direct child assessments, video recorded caregiver-child interactions, and administered caregiver questionnaires to evaluate child and caregiver outcomes. In addition, we measured children’s height and weight. The assessment took between 60 and 75 minutes. Data collectors and video coders were blinded to the group allocation to prevent assessment bias. The same procedure was repeated for all 3 assessment rounds.

Qualitative data were collected concurrently with the quantitative assessments at the 3 time points. Interviews and focus groups were conducted with a randomly selected sample of caregivers in both the index intervention and active control groups and with all DBS and PA facilitators. With caregivers, we aimed to explore their subjective assessment of the DBS or PA sessions, the actual adoption of DBS or PA practices in the home, and the perceived impact of the intervention on their children. Possible barriers to adopting DBS or PA practices at home were examined. A set of questions targeting their parenting attitudes and practices, asked in all 3 rounds of data collection, maps out the potential effects of the intervention in these areas. Focus groups with DBS and PA facilitators provided additional information on the perceived reactions of caregivers to the content of sessions and their engagement with the sessions’ content. Tanzanian team members trained and supervised by senior research team members conducted all the interviews and focus groups.

All quantitative and qualitative assessments were administered in Swahili. The schedule is presented in Table 3.

The primary, secondary, and exploratory outcome measures and moderators assessed by the EDBiT study are presented in subsequent sections and Table 4.

**Table 3:** Schedule of enrollment, interventions, and assessments.

Time point					T0^a^			T1^b^			II^c^			T2^d^			EI^e^			T3^f^
**Enrollment**																				
	Eligibility screening			✓																
	Informed consent			✓																
	Allocation			✓																
**Interventions**																				
	Index intervention group sessions									✓						✓				
	Active control group sessions									✓						✓				
	Passive control group sessions																			
**Quantitative assessments**																				
	**Primary outcomes**																			
		Child language				✓						✓						✓		
		Parental sensitivity and parent-child reciprocity				✓						✓						✓		
	**Secondary outcomes**																			
		Child attention				✓						✓						✓		
		Child behavior				✓						✓						✓		
		Parenting				✓						✓						✓		
		Parental stress				✓						✓						✓		
	**Exploratory outcomes**																			
		Child health				✓						✓						✓		
	**Moderators and selected confounding variables**																			
		Sex of the child	✓																	
		Socioeconomic situation	✓																	
		Cognitive abilities of the caregiver				✓														
	**Qualitative assessments**																			
		Interviews with caregivers				✓						✓						✓		
		Focus groups with caregivers										✓								
		Focus groups with DBS^g^ and PA^h^ facilitators										✓								

^a^T0: allocation and enrollment.

^b^T1: baseline.

^c^II: intensive intervention.

^d^T2: post–intensive intervention assessment.

^e^EI: extensive intervention.

^f^T3: post–extensive intervention assessment.

^g^DBS: dialogic book-sharing.

^h^PA: playful activity.

**Table 4:** Primary and secondary outcome measures.

Outcomes			Measures
**Primary outcomes**			
	Child language	Receptive and expressive language: MacArthur-Bates Communication Development Inventory (short-form version; adapted) Receptive language: Peabody Picture Vocabulary Test (adapted) Expressive language: Expressive One Word Picture Vocabulary Test (adapted)	
	Parental sensitivity and parent-child reciprocity	Parental sensitivity and parent-child reciprocity: directly observed book-sharing task rated with the Murray Global Rating Scale	
**Secondary outcomes**			
	Child attention	Early Child Vigilance Task	
	Child behavior	Prosocial behavior: help task Prosocial behavior: Strengths and Difficulties Questionnaire (prosocial subscale) Behavior: Strengths and Difficulties Questionnaire; total difficulties score	
	Parenting	Parental engagement with child: frequency of engagement in joint play with child, self-recorded in diaries Parenting practices: Parenting Styles and Dimensions Questionnaire (connection, physical coercion, and verbal hostility dimensions) Parenting efficacy: Parenting Self-Efficacy questionnaire (TOPSEa; selected questions)	
	Parental stress	Parental Stress Scale	
**Exploratory outcomes**			
	Child health	Incidence of diarrhea Wasting Stunting Underweight	
**Moderators and selected confounding variables**			
	Sex of the child	Child's sex (male or female)	
	Socioeconomic situation of families	Asset index	
	Parental cognitive abilities	Vienna Matrices Test	

^a^TOPSE: Tool to Measure Parenting Self-Efficacy.

#### Primary Outcomes

##### Child Language

Child language was assessed using a combination of direct child assessment and caregiver-reported measures. Caregivers reported on their children’s expressive and receptive language levels using the MacArthur-Bates Communication Development Inventory [34]. For this study, a short version with 55 words was used.

Two assessment tools were used to complement the MacArthur-Bates Communication Development Inventory with direct measures of child language. Language comprehension (receptive language) of all children was measured using an adapted version of the Peabody Picture Vocabulary Test [35] during which the child was shown pictures of 80 items in groups of 4. The child was asked to point to 1 named item among the 4 (eg, “Where is the goat?”), with the number of correct responses recorded.

Expressive language was only assessed in the group of children aged between 30 and 45 months, using the adapted Expressive One Word Picture Vocabulary Test [36]. The child was asked to say the name of the item shown on a screen. In total, 20 pictures were shown, and the number of correct responses was recorded.

All these instruments have been used in previous evaluations of book-sharing programs in African contexts in the same form as in our study [15,21-23,37]. To reflect the vocabulary to which local children in our study setting are typically exposed, we only adapted the set of words in each test based on numerous consultations. The instruments were piloted with 10 caregiver-child dyads.

##### Parental Sensitivity and Parent-Child Reciprocity

Parental sensitivity and parent-child reciprocity were assessed by direct observation of the caregiver and child sharing a book. The interaction was recorded on video and coded by independent assessors blinded to the group allocation using a coding system developed by LM, adapted for the book-sharing context. While specific modes of expressing sensitivity and reciprocity can vary between cultures [38,39], the core features are common across different populations, that is, supportive and warm responsiveness to infant cues and mutually positive active engagement. This coding system has been used previously in DBS studies, including the investigation of book-sharing impact in LMICs and middle-income settings in South Africa, Lesotho, Colombia, and Brazil. Notably, in one South African study, sensitivity and reciprocity were found to mediate the impact of the intervention on the development of child language and attention [21].

#### Secondary Outcomes

##### Child Attention

The Early Childhood Vigilance Task (ECVT) was used to assess child attention [40]. ECVT is a computer-based tool during which the child is shown a 7-minute-long cartoon. The child’s sustained attention is indexed by the number of seconds the child attends to the screen during the 7-minute period. The child was videotaped during the assessment, and the recording was subsequently independently scored. ECVT has been previously used in DBS studies [21,22].

##### Child Behavior

Child behavior was assessed using the full version of the Strengths and Difficulties Questionnaire [41], with aggregated scores obtained for the following scales: emotional symptoms, conduct problems, hyperactivity and inattention, relationship problems, and prosocial behavior as well as for a total score indexing total level of behavioral disturbance. The child’s prosocial behavior was further assessed during a help task [21,42]. In this direct assessment, the data collector creates a situation in which the child is encouraged to express a “helping behavior,” that is, to offer help in finding the item the data collector pretends to have lost.

##### Parenting

The frequency of caregivers joining the children in sharing a book at home and in other child games was assessed using diaries distributed to all caregivers in all groups. Caregivers were provided with an easy-to-fill-out diary for the 8-week intensive intervention period. Every day, caregivers circled pictograms of the child’s activities and the person or people who joined the child in that activity. Diaries were collected after the intensive intervention period.

To assess caregivers’ general parenting practices, 3 dimensions from the self-report short version of the Parenting Styles and Dimensions Questionnaire [43] were administered: the connection dimension to index the occurrence of warm and supportive behaviors, the physical coercion dimension to index the extent to which caregivers apply physical punishments, and the verbal hostility dimension to index the presence of harsh verbal behavior.

In total, 13 statements from the Tool to Measure Parenting Self-Efficacy questionnaire [44] were used to assess parenting efficacy concerning emotion and affection, play and enjoyment, empathy and understanding, control, discipline and setting boundaries, self-acceptance, and learning and knowledge.

##### Parental Stress

The Parental Stress Scale was administered to assess the level of parenting-related stress [45].

#### Exploratory Outcomes (Child Health)

To assess child health, caregivers reported whether the child experienced diarrhea in the past 2 weeks. In addition, children’s height and weight were measured to check their nutritional status (stunting, wasting, and underweight).

#### Potential Moderators

##### Sex of the Child

We examined the potential difference between the effects of the intervention on girls and boys.

##### Socioeconomic Situation of Families

We examined the potential difference in the intervention’s effects on children living in families of varying socioeconomic statuses using the asset index [46,47] as an indicator of socioeconomic status. The indicators recorded are defined based on the same taxonomy used, for example, in the Demographic and Health Surveys [48]. Following the procedures of Němečková et al [49], the individual items are weighted during aggregation using weights generated by the multiple correspondence analysis method, and the results are linearly rescaled to a 0 to 100 scale.

##### Cognitive Abilities of Caregivers

The shortened version of the Vienna Matrices Test [50] was administered to measure the cognitive abilities of caregivers.

### Fidelity Testing

Consistent delivery of both the DBS and PA programs was tracked with an 18-item implementation fidelity checklist. In total, 11 items assessed the substantive content of each session, for example, whether the facilitator explained key concepts clearly and gave accurate, supportive feedback during mothers’ practice, whereas 7 items captured organizational logistics and caregiver-oriented factors (eg, punctual arrival of facilitators and mothers, maternal attentiveness during the theoretical segment, distribution of porridge to children at the start of the session, and the babysitter’s behavior).

Each checklist item was transformed to a 0% to 100% scale; the mean of the 11 content items formed the content fidelity subscore, and the mean of the 7 organizational items formed the procedural fidelity subscore. A session was deemed adequate only when both subscores were ≥75%. Facilitators whose scores fell below this threshold received targeted supervisory feedback before the next block of sessions. Fidelity distributions will be summarized descriptively in trial reports.

An independent rater conducted live, in-person observations of 2 sessions per facilitator, with a minimum interval of 3 weeks between visits. Sessions were not audio or video recorded. Fidelity scores will be used solely to define the per-protocol analytic sample (dyads taught by facilitators whose average content and procedural fidelity across the 2 observed sessions are each ≥75%). Because the checklist serves primarily as a quality-assurance gate, fidelity will otherwise be summarized descriptively and will not be modeled as an explanatory variable.

### Data Management

Data were collected on tablets and mobile phones using a Kobo platform [51] and uploaded to a secure online server. Child assessments and caregiver-child book-sharing interactions were video recorded. A random sample of videos was regularly checked for fidelity of the data collection procedure. Interviews and focus groups were recorded on a voice recorder and checked for quality and adherence to the prescribed questions.

### Data Analysis

#### Statistical Analysis Plan

All data will be analyzed by an independent statistician using R (R Foundation for Statistical Computing) or Stata (StataCorp LLC). Fidelity will first be described with standard descriptive statistics. Hypothesis tests will be 2-sided with α of .05, and effect-size statistics will be reported to indicate the magnitude of any intervention effects.

Outcomes are classified a priori as primary, secondary, or exploratory. For the primary outcomes, we will use the Holm-Bonferroni method to adjust *P* values. Secondary and exploratory outcomes will be interpreted as hypothesis generating; the Benjamini-Hochberg procedure will be applied for their significance evaluation.

The primary and secondary outcome analyses will follow the intention-to-treat principle.

Linear mixed models or, where appropriate, analyses of covariance will evaluate intervention effects at both the intensive and extensive postintervention phases, always adjusting for baseline outcome scores. Because participants are clustered, a random effect will be included, and fixed-effect estimates will be compared with intercluster variability. Before modeling, every numerical predictor will be mean centered and standardized (*z* scores); interaction terms will be created as products of these centered variables. Candidate covariates and moderators (child age, dwelling place, child sex, socioeconomic situation, and parental cognitive abilities as indexed by education) will be retained only if they are significant or if removing them markedly alters other parameter estimates.

Although the intention-to-treat approach is the primary approach, a complementary per-protocol analysis will be performed to clarify whether any lack of impact arises from deficiencies in the intervention itself or from participants’ incomplete exposure to it.

Multicollinearity among predictors will be assessed using variance inflation factors, with appropriate mitigation strategies applied where necessary.

#### Mediator and Moderator Analyses

Mediator analysis will discern whether improvement in caregiver sensitivity mediates intervention effects on child outcomes. In addition, we will investigate whether improvements in parental sensitivity mediate advancements in reductions of parental stress. Mediation analyses will be conducted using the product-of-coefficients approach (a series of path models within the mixed-effects framework). CIs will be obtained using bootstrapping.

To assess moderation, interaction terms between the intervention and possible moderators will be included in the model. Subsequently, the significance of the interaction will be tested. In moderator analyses, we will also conduct a subgroup investigation to identify variations in the differential effectiveness of the intervention across subgroups. Subgroup analyses will be conducted with respect to the number of sessions attended, child sex, living standard, and the cognitive abilities of the caregiver.

#### Statistical Power

The required sample size was calculated according to accepted mathematical principles (48,49].

The sample size in each of the 3 arms of the study was computed using the following formula:




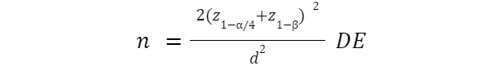




where α of .05 is the significance level, ...1–β (.80) is the power of the test, and *z*_1–α/4_ (2.24) and z_1–β_ (0.84) are quantiles of the standard normal distribution. The significance level α of .05 is divided by 4—the number of comparisons performed while primary hypotheses are tested (in fact, this is the so-called Bonferroni correction). All tests have 1-sided alternatives. On the basis of the previous meta-analytic studies [52,53], power is guaranteed for the effect size of 0.5. Finally, *DE* is the so-called design effect. It takes into account the clustered nature of data and is computed using the following formula:

DE = 1 + ([cv2 + 1]m –1)ICC

where the *m* value of 3 is the expected average cluster size, *ICC* is the intracluster correlation coefficient, and *cv* is the coefficient of variation of the cluster size. We set the *IC*C at 0.05—more than twice the commonly reported value of approximately 0.02 in comparable parenting studies. This higher value inflates the design effect and, in turn, the required sample size, providing a buffer that preserves statistical power. The value of *cv*^2^=2/9 was used, corresponding to a discrete uniform distribution of cluster sizes from 1 to 5. This range reflects both the settlement layout and our clustering procedure, which aimed to retain as many eligible households as possible while keeping clusters compact (refer to the Eligibility, Recruitment, and Randomization section). Substituting these values into the formula mentioned earlier, one obtains a *DE* of 1.13.

Taking into account a potential attrition rate of up to 15%, the required sample size of each arm is 100. Observations with missing values of the outcome variable will be excluded from the analysis that deals with the given outcome but could still be included in other analyses. Missing values in covariates will be substituted by their regression estimates based on other covariates. The impact of the substitution will be discussed by comparing the current estimates with those obtained if the observations with missing values were excluded.

#### Qualitative Data Analysis

Qualitative interviews and focus group recordings were transcribed verbatim and translated by a native Swahili speaker fluent in English. The transcriptions will be analyzed by applying thematic analysis. Deductive and inductive coding will be used to capture the variety of information in the data. The codes will then be grouped into themes, capturing the recurring patterns in the data. Some codes may become themes on their own. Consequently, the themes will be reviewed against the data corpus. The ATLAS.ti software (Lumivero) will be used for data organization and structuring through managing and visualizing the developed codes’ and themes’ system. More than one researcher will perform the data analysis and interpretation to provide depth to the process. The researchers will regularly discuss the coding scheme to ensure consistency and reliability.

#### Mixed Methods Data Integration

Qualitative data hold the potential to explain key outcomes of the quantitative analysis. To maximize the explanatory potential of our mixed methods study, we will (1) integrate quantitative and qualitative datasets in an interpretative joint display, (2) use the articulated ToC (Figure 1) as an integrative analytical framework, and (3) provide a narrative integration in the Discussion section. First, the joint display matrix will present quantitative findings alongside representative qualitative themes and illustrative quotes from interviews and focus groups. The matrix will also include a section for the interpretation of data, discussing possible convergence, divergence, or expansion of understanding emerging from integrating quantitative and qualitative datasets. Second, we will align the findings with our ToC components. Quantitative data will confirm or disprove the achievement of the expected outcomes presented in the ToC, while qualitative data will shed light on the reasons why and how these outcomes were or were not achieved and how the contextual factors shaped the intervention’s impact. Integrating these findings will allow us to revise and refine the original ToC. Finally, the Discussion section will explore the intersection between our quantitative and qualitative findings, providing a comprehensive assessment of the intervention’s outcomes and illuminating the mechanisms of change.

## Results

The data collection for the EDBiT study was completed in September 2024. The research team proceeds with analyzing the data collected in the 3 rounds of data collection. The study results are expected to be published by late 2025.

## Discussion

### Principal Aims and Expected Contributions

The EDBiT trial addresses a critical evidence gap—whether DBS can enhance early language, socioemotional competence and caregiver behavior in resource-constrained, rural settings in sub-Saharan Africa. By comparing DBS with both a WLC group and an active PA control group, the study will isolate benefits specific to DBS rather than the generic advantages of increased parent-child interaction. Its mixed methods design, underpinned by an articulated ToC, will also illuminate how and for whom DBS works, thereby advancing implementation science for scalable parenting interventions.

### Strengths and Limitations

Most RCTs of book sharing have been conducted in high-income or urban settings and have rarely included an active control group, leaving open the question of whether the gains stem from DBS itself or simply from extra parent-child time. EDBiT also helps close this gap by testing the intervention in rural Tanzania, where material resources are limited. In addition, this study’s mixed methods approach brings quantitative impact estimates into dialogue with caregivers’ lived experiences, generating implementation insights that purely quantitative trials often miss.

Nevertheless, inevitable field-trial constraints remain. The findings may not be readily generalizable to other locations or time points, and reliance on self-report for some caregiver outcomes could introduce social-desirability bias despite triangulation with observational data.

### Future Directions

If DBS intervention proves effective, future work could explore delivery to other household members, such as older siblings and fathers. Digital adaptations might be tested where printed materials are scarce. In addition, future work might experiment with tailoring program content or dosing for distinct subgroups, for example, mothers with lower cognitive ability or those living in extreme poverty, to determine whether customized approaches enhance the interest of mothers and intervention impact.

### Dissemination Plan

Findings will be shared with (1) local stakeholders in the Mbeya region of Tanzania through community forums and briefing sessions and (2) the global research community through presentations at international early-childhood conferences and open-access publications in peer-reviewed journals. Anonymized datasets will also be deposited in an open repository.

## Abbreviations

DBS: dialogic book-sharing
ECVT: Early Childhood Vigilance Task
EDBiT: Effects of a Dialogic Booksharing Intervention for Female Caregivers in Rural Tanzania
LMICs: low- and middle-income countries
PA: playful activity
RCT: randomized controlled trial
ToC: theory of change
WLC: waitlist control

## Data Availability

The full checklist with its scoring rubric is available from the authors on request.
